# Transseptal puncture and catheter ablation of atrial fibrillation in patients with atrial septal occluder devices: procedural techniques and outcomes

**DOI:** 10.1093/europace/euag071

**Published:** 2026-04-07

**Authors:** Kodai Saito, Koji Nakagawa, Mitsutaka Nakashima, Takuro Masuda, Rie Nakayama, Akira Ueoka, Takashi Miki, Saori Asada, Yoichi Takaya, Norihisa Toh, Nobuhiro Nishii, Teiji Akagi, Hiroshi Morita, Takayuki Inomata, Shinsuke Yuasa

**Affiliations:** Department of Cardiovascular Medicine, Okayama University Graduate School of Medicine, Dentistry and Pharmaceutical Sciences, 2-5-1 Shikata-Cho, Kiaku, Okayama 700-8558, Japan; Department of Cardiovascular Medicine, Niigata University Graduate School of Medical and Dental Sciences, Niigata, Japan; Department of Cardiovascular Medicine, Okayama University Graduate School of Medicine, Dentistry and Pharmaceutical Sciences, 2-5-1 Shikata-Cho, Kiaku, Okayama 700-8558, Japan; Department of Cardiovascular Medicine, Okayama University Graduate School of Medicine, Dentistry and Pharmaceutical Sciences, 2-5-1 Shikata-Cho, Kiaku, Okayama 700-8558, Japan; Department of Cardiovascular Medicine, Okayama University Graduate School of Medicine, Dentistry and Pharmaceutical Sciences, 2-5-1 Shikata-Cho, Kiaku, Okayama 700-8558, Japan; Department of Cardiovascular Medicine, Okayama University Graduate School of Medicine, Dentistry and Pharmaceutical Sciences, 2-5-1 Shikata-Cho, Kiaku, Okayama 700-8558, Japan; Department of Cardiovascular Medicine, Okayama University Graduate School of Medicine, Dentistry and Pharmaceutical Sciences, 2-5-1 Shikata-Cho, Kiaku, Okayama 700-8558, Japan; Department of Cardiovascular Medicine, Okayama University Graduate School of Medicine, Dentistry and Pharmaceutical Sciences, 2-5-1 Shikata-Cho, Kiaku, Okayama 700-8558, Japan; Department of Cardiovascular Medicine, Okayama University Graduate School of Medicine, Dentistry and Pharmaceutical Sciences, 2-5-1 Shikata-Cho, Kiaku, Okayama 700-8558, Japan; Department of Cardiovascular Medicine, Okayama University Graduate School of Medicine, Dentistry and Pharmaceutical Sciences, 2-5-1 Shikata-Cho, Kiaku, Okayama 700-8558, Japan; Department of Cardiovascular Medicine, Okayama University Graduate School of Medicine, Dentistry and Pharmaceutical Sciences, 2-5-1 Shikata-Cho, Kiaku, Okayama 700-8558, Japan; Department of Cardiovascular Therapeutics, Okayama University Graduate School of Medicine, Dentistry and Pharmaceutical Sciences, Okayama, Japan; Department of Cardiovascular Medicine, The Sakakibara Heart Institute of Okayama, Okayama, Japan; Department of Cardiovascular Therapeutics, Okayama University Graduate School of Medicine, Dentistry and Pharmaceutical Sciences, Okayama, Japan; Department of Cardiovascular Medicine, Niigata University Graduate School of Medical and Dental Sciences, Niigata, Japan; Department of Cardiovascular Medicine, Okayama University Graduate School of Medicine, Dentistry and Pharmaceutical Sciences, 2-5-1 Shikata-Cho, Kiaku, Okayama 700-8558, Japan

**Keywords:** Atrial septal occluder, Transseptal puncture, Atrial fibrillation, Catheter ablation, Cardiac computed tomography

## Abstract

**Aims:**

Transseptal puncture (TSP) for left atrial (LA) access during catheter ablation poses technical challenges in patients with an atrial septal occluder (ASO) device because of altered septal anatomy and device coverage. However, detailed procedural techniques remain insufficiently described. This study aimed to describe a practical, case-based approach to TSP in patients with ASO devices and to evaluate procedural outcomes.

**Methods:**

Between March 2005 and September 2025, a total of 1550 patients underwent ASO device implantation at our institution. Among them, 28 consecutive patients who later underwent atrial fibrillation (AF) ablation between March 2017 and September 2025 were retrospectively analysed. A representative clinical case illustrates the procedural workflow.

**Results:**

Transseptal access was successfully achieved in 27 of 28 patients (96.4%). TSP was performed through the native septum in 17 patients and through the ASO device in 11 patients. Balloon dilation was frequently required in device puncture cases. Pulmonary vein isolation and adjunctive ablation were successfully completed in all patients without major complications. Small residual interatrial shunts observed immediately after the procedure resolved spontaneously during follow-up.

**Conclusion:**

TSP and AF ablation can be performed safely and effectively in patients with ASO devices. A case-based procedural approach with appropriate imaging guidance enables reliable LA access even in the presence of an ASO device.

## Graphical Abstract

**Graphical Abstract:**
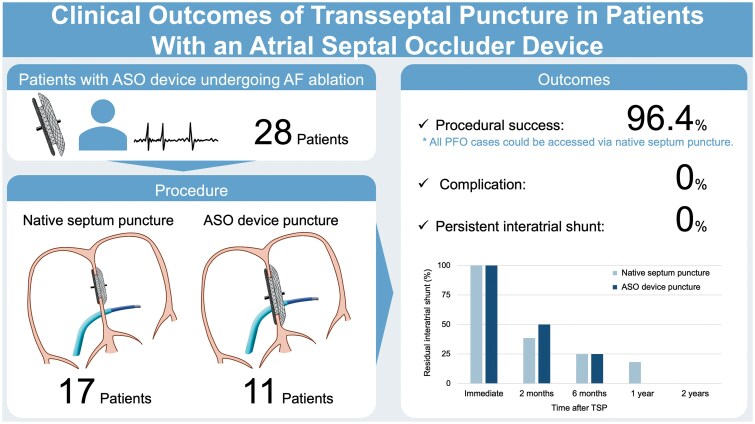
AF, atrial fibrillation; ASO, atrial septal occluder; PFO, patent foramen ovale; TSP, transseptal puncture

What’s new?

- This study provides a detailed, step-by-step description of transseptal puncture through atrial septal occluder devices, illustrated by a representative clinical case.
- A key technical insight is the deflate-and-dive technique, enabling smooth sheath passage across the recoiling nitinol mesh immediately after balloon dilation.
- Long-term follow-up demonstrates spontaneous resolution of residual interatrial shunts without haemodynamic significance.
- These findings support the safety and feasibility of transseptal puncture and catheter ablation of atrial fibrillation in patients with an atrial septal occluder device.

## Introduction

Patients with atrial septal defects (ASD) are at increased risk of developing atrial fibrillation (AF), and this risk persists even after ASD closure.^1–4^ Previous reports, including our own, have demonstrated that catheter ablation of AF prior to ASD closure can reduce the risk of recurrence.^5^ However, AF still recurred in 21% of such cases, and other studies have reported that 14.9% of patients developed new-onset AF after ASD closure during follow-up.^6^ In addition to ASD, patent foramen ovale (PFO) closure is increasingly performed, particularly in patients with cryptogenic stroke.^7^ While PFO itself does not directly predispose to AF, new-onset AF following PFO closure has emerged as a relevant clinical concern. Moreover, even in patients initially referred for paradoxical embolism, the risk of developing AF increases with age.^8^

As the number of percutaneous atrial septal closure procedures continues to rise—whether for ASD or PFO—the population of patients with an atrial septal occluder (ASO) device who develop or recur AF is expected to grow. Catheter ablation remains an effective treatment for AF, but transseptal puncture (TSP) in patients with ASO devices presents technical challenges due to altered septal anatomy and device coverage.

Although some case series and reports have described TSP after ASO device implantation,^9–12^ detailed procedural techniques—particularly involving direct ASO device puncture—and long-term outcomes have been scarcely reported. Therefore, the aim of this study was to provide a case-based, step-by-step procedural description of TSP through ASO devices, highlighting key technical aspects and procedural tips, and to report outcomes of catheter ablation in this population.

## Methods

### Study population

From March 2005 to September 2025, a total of 1550 patients underwent ASO device implantation at Okayama University Hospital. Among them, 28 consecutive patients who underwent AF/AT ablation after ASO device implantation were included. The study was conducted in accordance with the Declaration of Helsinki and approved by the institutional ethics committee of Okayama University Hospital (approval number: K2412-044). The requirement for informed consent was waived because of the low-risk nature of the study and because consent could not be directly obtained from all enrolled patients.

### Preprocedural preparation

All procedures were performed under conscious sedation or general anaesthesia, with continuous monitoring of vital signs. Long-term anticoagulation with warfarin or direct oral anticoagulants (DOACs) was maintained throughout the periprocedural period without interruption. Patients received intravenous unfractionated heparin after venous puncture but prior to TSP. During the procedures, the target activated clotting time (ACT) was maintained between 300 and 400 s.^13–15^

### Selection of puncture site

**Figure 1:**
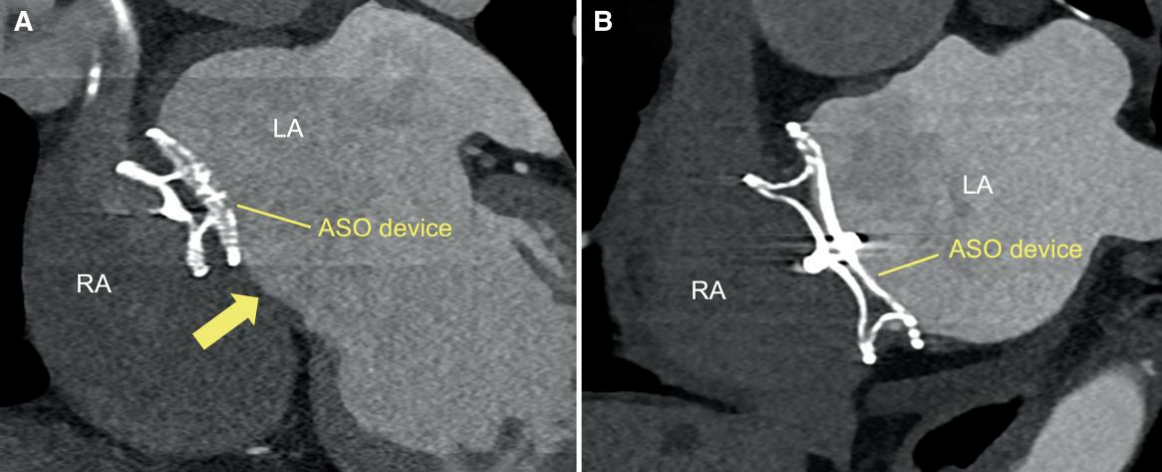
Cardiac computed tomography assessment of native septum for transseptal puncture. (*A*) Cardiac computed tomography image showing the infero-posterior puncturable native septum (arrow) adjacent to the ASO device. (*B*) Cardiac computed tomography image showing the ASO device covering most of the interatrial septum, leaving no identifiable puncturable native septum. ASO, atrial septal occluder.

All patients underwent cardiac computed tomography (CCT) and transoesophageal echocardiography (TEE) for procedural planning. CCT was primarily used to assess septal anatomy and to select the TSP site. TEE was performed before the procedure to exclude left atrial (LA) thrombus and confirm ASO device position. If a puncturable native septum was present beside the ASO device (*Figure 1A*), the native septum was targeted—typically the inferoposterior portion—while anterior or superior puncture sites were avoided due to the difficulty of ablation procedures, including pulmonary vein isolation, and the potential risk of aortic injury or atrioventricular block. If the ASO device completely covered the interatrial septum (*Figure 1B*), or after an unsuccessful attempt to puncture the native septum, direct puncture of the ASO device was performed. Intracardiac echocardiography (ICE) was used in all sessions to provide real-time visualization and confirm whether a safe native septal area was present, as well as to guide fine adjustments of catheter orientation before puncture.

### Optimal puncture area on the ASO device

**Figure 2:**
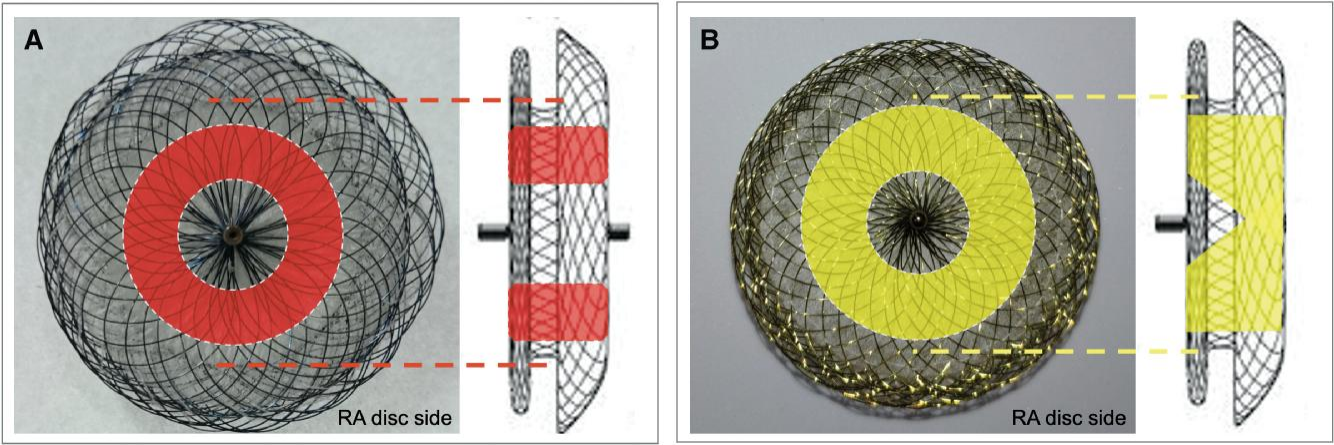
Optimal puncture areas on two types of atrial septal occluder devices. Schematic illustrations comparing the structural configuration of the Amplatzer Septal Occluder (*A*) and the Figulla Flex II Septal Occluder (*B*). In both devices, the optimal puncture area is indicated by the shaded region, corresponding to the off-centre waist where the nitinol mesh is relatively sparse and the number of layers to traverse is minimized. In the FFII device, the hubless left atrial disc allows a broader safe zone, whereas in the Amplatzer device, the central hub and denser mesh restrict the feasible puncture area to a narrower off-centre region.

The waist portion of the ASO device, specifically slightly off-centre from the hub, was considered optimal for TSP. The central hub has dense metal structures, making direct penetration challenging, whereas the off-centre waist region has a relatively sparse nitinol mesh, facilitating easier passage of the needle (*Figure* *2A* and *B*). Puncturing through the disc portion would require traversing multiple layers (RA disc, native septum, LA disc), increasing resistance. By targeting the waist region, access could be gained through only two layers, enhancing procedural safety.

### Representative case (ASO device puncture)

The representative case was a 66-year-old woman with symptomatic AF who had undergone transcatheter ASD closure using a 36-mm Amplatzer Septal Occluder 10 years earlier. Preprocedural CCT and TEE suggested no adequate puncturable native septum adjacent to the device, and direct ASO device puncture was planned.

#### Step 1. Fluoroscopy setup and landmarking

**Figure 3:**
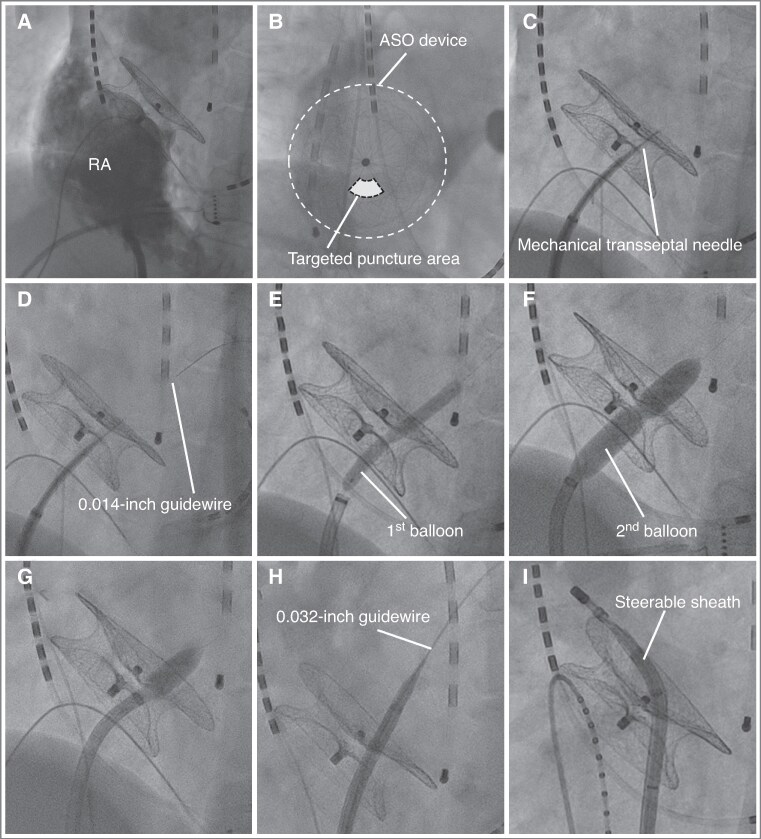
Step-by-step procedural sequence of TSP through an ASO device. (*A*) RA angiography (LAO fluoroscopic view) delineates the interatrial septum and the ASO device relationship. (*B*) The optimal puncture area is located at the 5–7 o’clock position on the device under the RAO fluoroscopic view (shaded area). (*C*) A puncture is performed using a mechanical transseptal needle inserted into an SL-0 sheath. (*D*) After confirming LA entry by contrast injection, a 0.014-inch guidewire is advanced into the LA. If the tip of the inner dilator in the sheath can be advanced into the LA at this point, the needle and 0.014-inch guidewire should be exchanged for a 0.032-inch guidewire. (*E*) Initial dilation is performed using a 4–5 mm balloon. (*F*) If the sheath cannot penetrate the ASO device, balloon is upsized to 6–8 mm. (*G*) The sheath is advanced into the LA while the balloon is being deflated (the deflate-and-dive technique). (*H*) Using a 0.032-inch guidewire, the SL-0 is exchanged for a steerable sheath. (*I*) Then, an ablation catheter is inserted into the steerable sheath, and the ablation procedure is initiated. ASO, atrial septal occlude; LA, left atrium; LAO, left anterior oblique; RA, right atrium; RAO, right anterior oblique; TSP, transseptal puncture.

A biplane fluoroscopic system with right anterior oblique (RAO) and left anterior oblique (LAO) views was used. The RAO projection was adjusted to obtain an en face view of the ASO device, whereas the LAO projection was slightly adjusted to achieve a tangential view of the device. Right atrial (RA) angiography was performed to visualize the anatomical relationship between the atrial septum and ASO device (*Figure 3A*, Supplementary material online, *Video S1*).

#### Step 2. Sheath positioning on the RA side of the device

A Swartz™ SL-0 sheath (8.5 Fr, 63 cm, Abbott) was advanced to the RA surface of the ASO device at the defined puncture area. For procedural stability and catheter manoeuverability during ablation, the puncture was directed towards the 5–7 o’clock position on the device under the RAO fluoroscopic view, corresponding to the lower part of the optimal puncture area (*Figure 3B*).

#### Step 3. Device puncture using a mechanical needle

**Figure 4:**
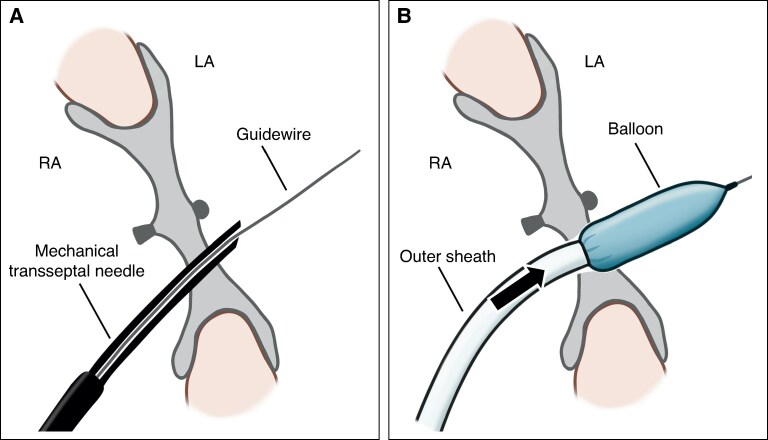
Key technical steps for transseptal puncture through an ASO device. (*A*) Use of a mechanical needle: A mechanical needle with a distal tip hole was used to enable passage of a 0.014-inch guidewire for balloon delivery through the needle lumen. (*B*) Deflate-and-dive technique: After septal dilation with a balloon, the outer sheath is advanced across the septum in synchrony with balloon deflation to minimize resistance and prevent mesh recoil. ASO, atrial septal occluder; LA, left atrium; RA, right atrium.

The mechanical transseptal needle (non-radiofrequency needle) was advanced perpendicularly until a loss of resistance was felt (*Figure 3C*, Supplementary material online, *Video S2A* and *S2B*). Contrast was injected to confirm LA entry, followed by careful advancement of a 0.014-inch guidewire (*Figure 3D*, Supplementary material online, *Video S3*). Unlike radiofrequency needles, the mechanical needle has a distal tip lumen that allows immediate passage of a guidewire into the LA after puncture. This enables subsequent balloon delivery (*Figure 4A*).

#### Step 4. Balloon dilation to enable dilator/sheath passage

The SL-0 inner dilator did not advance spontaneously, as was observed in most cases. Therefore, the balloon dilation with a 4-mm balloon was performed to facilitate advancement of the inner dilator into the LA (*Figure 3E*, Supplementary material online, *Video S4*). The outer sheath could not be advanced after the initial dilation, a second balloon dilation with an 8-mm balloon was performed to further enlarge the tract (*Figure 3F*).

#### Step 5. Deflate-and-dive technique

Because the nitinol mesh of the ASO device recoils immediately after balloon dilation, the puncture tract rapidly shrinks and the edge of the outer sheath may catch on the mesh, making sheath advancement difficult. To overcome this problem, we applied the deflation-and-dive technique, in which the outer sheath is advanced precisely in synchrony with balloon deflation (*Figure 3G* and *4B*, Supplementary material online, *Video S5*). By allowing the sheath to ‘dive’ through the tract at the moment of balloon deflation, this manoeuver maintains tract patency and facilitates smooth sheath passage across the device mesh.

#### Step 6. Wire exchange and sheath optimization

Once the inner dilator reached the LA, the guidewire was exchanged for a 0.032-inch wire to provide additional support (*Figure 3H*). After the SL-0 sheath was advanced into the LA, it was replaced with a steerable sheath, as torque control was limited in ASO device-puncture cases (*Figure 3I*, Supplementary material online, *Video S6*).

#### Step 7. Catheter ablation in the LA

**Figure 5:**
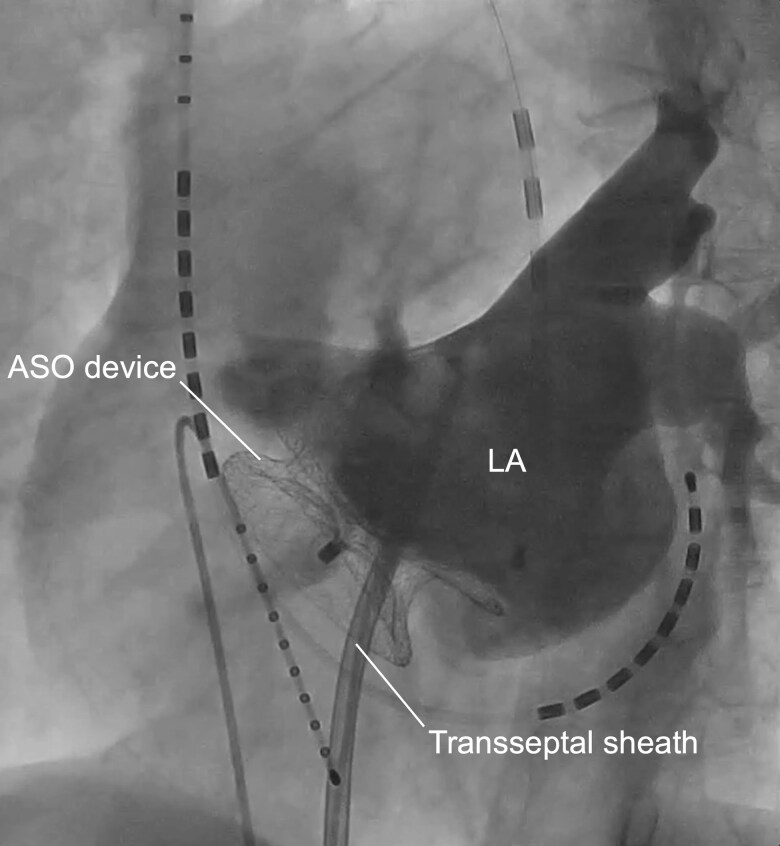
Left atrial angiography. LA angiography demonstrating pulmonary vein anatomy and the spatial relationship between the pulmonary veins and the ASO device. See Supplementary material online, *Video S7*. LA, left atrium; ASO, atrial septal occluder.

**Figure 6:**
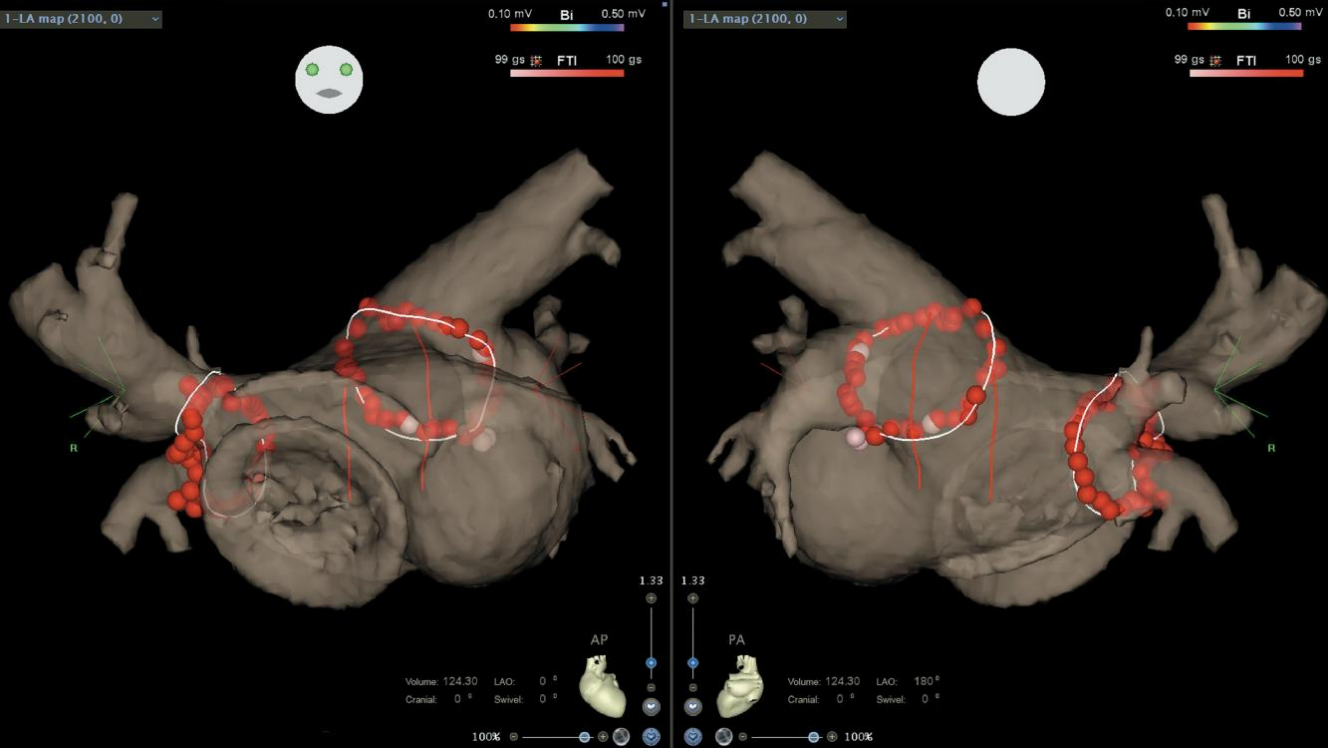
Pulmonary vein isolation line. The ablation line created along the pulmonary vein antrum. The LA disc of the ASO device interrupted the intended ablation line, requiring the anterior isolation line of the right pulmonary veins to be placed more distally. LA, left atrium; ASO, atrial septal occluder.

After LA access was achieved, LA angiography was performed to assess pulmonary vein anatomy and its relationship to the ASO device (*Figure 5*, Supplementary material online, *Video S7*). Pulmonary vein isolation was then performed using radiofrequency energy with SmartTouch SF (Biosense Webster, CA, USA). Because the LA disc of the ASO device interrupted the intended ablation line, the isolation line on the anterior aspect of the right pulmonary veins had to be placed more distally towards the pulmonary veins than the optimal line (*Figure 6*).

### Data collection and outcome measures

Clinical, procedural, and imaging data were retrospectively collected. Procedural parameters included TSP success rate, total procedure time, fluoroscopy time, and time from femoral puncture to LA access. Successful TSP was defined as confirmed LA access without major complications.

### Follow-up

All patients were followed through outpatient visits or by referring physicians. Evaluations included clinical assessment, ECG, and ambulatory monitoring when indicated. AF recurrence was defined as any documented AF episode requiring clinical attention.^16^ Residual interatrial shunts were routinely evaluated by TTE immediately after the procedure. Patients with a detected shunt underwent follow-up TTE, and in some cases, follow-up was concluded once shunt resolution was confirmed. Major cardiovascular events including thromboembolism or device-related complications were also monitored.

### Statistical analysis

Continuous variables are presented as median (IQR) and compared using the Mann–Whitney *U* test after normality assessment (Shapiro–Wilk). Categorical variables were compared using the *χ*^2^ or Fisher’s exact test, as appropriate. Time-to-event outcomes were analysed with Kaplan–Meier estimates and the log-rank test. ROC analysis was performed to assess discrimination for device puncture, and the optimal cutoff was determined using the Youden index. In one patient with a PFO in whom the RA disc size exceeded the LA disc size, the RA disc diameter was used for the analysis. All tests were two-sided with a significance level of 0.05. Analyses were performed in R (v4.5.0). Missing data were handled by complete-case analysis, and no multiplicity adjustment was applied given the exploratory nature of the study.

## Results

### Patient characteristics

Baseline characteristics of the 28 included patients are summarized in *Table 1*. The median age at ablation was 67.0 years (IQR 57.0–77.5), and the median interval between ASO device implantation and ablation was 7.0 years (IQR 3.0–8.0). Prior AF/AT ablation before ASO device implantation was seen in 53.6% of patients. Paroxysmal AF/AT was observed in 57.1% of patients. Comorbidities included hypertension (50.0%), dyslipidaemia (42.9%), diabetes mellitus (14.3%), and prior stroke (10.7%). All patients were receiving anticoagulation therapy.

**Table 1:** Baseline characteristics

Variables	*n* = 28
Age at ablation, years	67.0 (57.0–77.5)
Age at ASO device implantation, years	62.0 (52.5–69.5)
Interval between ASO device implantation and ablation, years	7.0 (3.0–8.0)
Male, *n*	13 (46.4)
Body mass index, kg/m^2^	23.1 (21.0–25.8)
Body surface area, m^2^	1.6 (1.4–1.7)
CHADS_2_ score (0/1/2/3/4/5/6), *n*	5/7/8/3/5/0/0
AF/AT ablation prior to ASO device implantation, *n*	15 (53.6)
Arrhythmia type, *n*	
Paroxysmal AF/AT	16 (57.1)
Persistent AF/AT	12 (42.9)
Comorbidities, *n*	
Hypertension	14 (50.0)
Dyslipidaemia	12 (42.9)
Diabetes mellitus	4 (14.3)
Coronary artery disease	0 (0)
Stroke	3 (10.7)
Anticoagulant, *n*	28(100)
DOAC	24 (85.7)
Warfarin	4 (14.3)
Antiplatelet, *n*	4 (14.3)

Data are expressed as median (interquartile range) or *n* (%).

Abbreviations: AF, atrial fibrillation; ASO, atrial septal occluder; AT, atrial tachycardia; DOAC, direct oral anticoagulant.

Structural and ASO device-related characteristics by puncture site are shown in *Table 2*. TSP was performed through the native septum in 17 patients and through the ASO device in 11 patients. ASO device-puncture cases had larger ASD diameters (29.0 [20.0–31.0] mm vs. 16.0 [11.0–19.8] mm, *P* = 0.024) and larger LA disc diameters (46.0 [38.0–50.0] mm vs. 31.5 [27.0–36.0] mm, *P* = 0.004). Posterior, IVC, or multiple rim deficiencies were observed only in the ASO device-puncture cases (53.6% vs. 0%, *P* < 0.001), whereas aortic rim deficiency was more frequent in the native septum-puncture cases (78.6% vs. 18.2%, *P* = 0.024). Echocardiographic parameters, including LA size and LVEF, were comparable.

**Table 2:** Structural features, device characteristics, and echocardiographic measurements

	Overall	Native septum-puncture group	ASO device-puncture group	
Variables	*n* = 28	*n* = 17	*n* = 11	*P* value
Diagnosis, *n*				
ASD	25 (89.3)	14 (82.4)	11 (100)	0.258
Maximum diameter, mm	19.0 (11.0–25.0)	16.0 (11.0–19.8)	29.0 (20.0–31.0)	0.024
Qp/Qs	2.2 (1.7–2.7)	2.1 (1.8–2.5)	2.7 (1.8–2.9)	0.272
Deficient rim, *n*				
None (sufficient rim)	5 (20.0)	3 (21.4)	2 (18.2)	1.000
Aortic rim	13 (52.0)	11 (78.6)	2 (18.2)	0.024
Posterior, IVC or multiple rim	7 (28.0)	0 (0.0)	7 (53.6)	<0.001
PFO	3 (10.7)	3 (17.6)	0 (0)	0.258
ASO device type, *n*				
Amplatzer Septal Occluder	18 (64.3)	10 (58.8)	8 (72.7)	0.689
Amplatzer Cribliform	2 (7.1)	2 (11.8)	0 (0)	0.505
Amplatzer PFO occluder	1 (3.6)	1 (5.9)	0 (0)	1.000
Figulla Flex Ⅱ Septal Occluder	6 (21.4)	3 (17.6)	3 (27.3)	0.653
Gore Cardioform ASD Occluder	1 (3.6)	1 (5.9)	0 (0)	1.000
LA disc diameter, mm	35.0 (30.0–42.5)	31.5 (27.0–36.0)	46.0 (38.0–50.0)	0.004
Echocardiography				
LA diameter, mm	43.0 (40.5–46.0)	43.5 (40.8–46.2)	42.0 (40.0–43.5)	0.334
LA volume index, mL/m^2^	46.0 (38.5–59.0)	47.0 (39.5–64.5)	45.0 (37.0–57.0)	0.905
LVEF, %	67.0 (59.5–70.0)	63.0 (59.0–67.8)	69.0 (68.5–70.0)	0.181

Data are expressed as median (interquartile range) or *n* (%). Continuous variables were compared using the Mann–Whitney *U* test, and categorical variables using Fisher’s exact test.

Abbreviations: ASD, atrial septal defect; ASO, atrial septal occluder; IVC, inferior vena cava; LA, left atrium; LVEF, left ventricular ejection fraction; PFO, patent foramen ovule.

ROC analysis showed good discrimination for predicting device puncture using the LA disc diameter (AUC 0.83, 95% CI 0.66–1.00). The optimal LA disc cutoff was 35 mm, with a sensitivity of 0.82 and specificity of 0.71, as shown in *Table 3*.

**Table 3:** Cutoff value of LA disc diameter for predicting the need for device puncture

Parameter	AUC (95% CI)	Optimal cutoff	Sensitivity	Specificity
LA disc diameter, mm	0.83 (0.66–1.00)	35 mm	0.82	0.71

Abbreviations are as in *Table 1*.

The optimal cutoff was determined by maximizing the Youden index. In one PFO case, the RA disc diameter was used for analysis because it was larger than the LA disc diameter.

### Procedural outcomes

Procedural outcomes are summarized in *Table 4*. TSP was successful in 27 patients (96.4%). One case with a 15-mm Figulla Flex II device (LA disc diameter: 30 mm), in which TSP through the device was required, failed due to inability to advance sheath or balloon. A single crossing sheath was used more often in ASO device-puncture cases (100% vs. 52.9%, *P* = 0.012); two sheaths were used only in native septum-puncture cases (47.1% vs. 0%, *P* = 0.010). Balloon dilation was required exclusively in ASO device-puncture cases (80.0% vs. 0%, *P* < 0.001). Median balloon size was 7.5 mm (IQR 7.0–8.0). Two patients in the ASO device-puncture cases achieved sheath passage without balloon dilation.

**Table 4:** Comparison of procedural characteristics

Variables	Overall	Native septum-puncture group	ASO device–puncture group	*P* value
	*n* = 28	*n* = 17	*n* = 11	
Puncture success, *n* (%)	27 (96.4)	17 (100)	10 (90.9)	0.393
Number of crossing sheath, *n* (%)	*n* = 27	*n* = 17	*n* = 10	
1 sheath	19 (70.4)	9 (52.9)	10 (100)	0.012
2 sheaths	8 (29.6)	8 (47.1)	0 (0.0)	0.010
Steerable sheath	25 (92.6)	17 (100.0)	8 (80.0)	0.128
Balloon dilation, *n* (%)	8 (29.6)	0 (0)	8 (80.0)	<0.001
Balloon diameter, mm	7.5 (7.0–8.0)	NA	7.5 (7.0–8.0)	NA
Time required for each procedure, min				
Total procedure time	211.0 (158.0–254.0)	200.0 (140.0–256.0)	218.0 (186.0–249.0)	0.312
Skin to LA (wire) time	19.5 (14.0–48.0)	15.0 (12.0–42.0)	27.0 (21.0–50.0)	0.145
Skin to LA (sheath) time	27.5 (17.0–57.0)	19.0 (16.0–32.0)	57.0 (45.0–99.0)	0.007
LA (wire) to LA (sheath) time	4.0 (3.0–8.8)	3.0 (3.0–4.0)	9.0 (7.0–28.0)	0.004
LA dwell time	153.5 (124.0–198.0)	155.0 (121.0–186.0)	145.0 (133.0–202.0)	0.936
Fluoroscopy time	21.5 (12.2–27.5)	20.0 (9.0–23.0)	47.0 (22.5–66.5)	0.016
Ablation procedures in the LA, *n* (%)				
Pulmonary vein isolation (radiofrequency)	19 (70.4)	12 (70.6)	7 (70.0)	1.000
Pulmonary vein isolation (pulsed-field)	1 (3.7)	1 (5.9)	0 (0)	1.000
Posterior wall isolation	7 (25.9)	3 (17.6)	4 (40.0)	0.365
LA focal ablation	9 (33.3)	6 (35.3)	3 (30.0)	1.000
Mitral isthmus linear ablation	6 (22.2)	4 (23.5)	2 (20.0)	1.000
Completion of procedure, *n* (%)	27 (100)	17 (100.0)	10 (100.0)	NA
Procedural complication, *n* (%)	0 (0)	0 (0)	0 (0)	NA

Abbreviations are as in *Tables 1* and *2*.

Total procedure time did not differ significantly according to the puncture site, although it tended to be longer in the ASO device-puncture cases (200 [140–256] min vs. 218 [186–249] min; *P* = 0.312). When individual procedural steps were examined, LA access was longer in ASO device–puncture cases: skin-to-LA (wire) 27.0 (21.0–50.0) min vs. 15.0 (12.0–42.0) min (*P* = 0.145); skin-to-LA (sheath) 57.0 (45.0–99.0) min vs. 19.0 (16.0–32.0) min (*P* = 0.007); and LA wire-to-sheath 9.0 (7.0–28.0) min vs. 3.0 (3.0–4.0) min (*P* = 0.004). LA dwell time did not differ between groups (145 [133–202] min vs. 155 [121–186] min; *P* = 0.936). Fluoroscopy time was longer in ASO device-puncture cases (47.0 vs. 20.0 min, *P* = 0.016).

Regarding LA ablation procedures, pulmonary vein isolation was the most frequently performed lesion (70.4%), followed by focal ablation (33.3%), posterior wall isolation (25.9%), and mitral isthmus ablation (22.2%). All planned ablations were completed, and no major complications occurred.

### Follow-up outcomes

**Figure 7:**
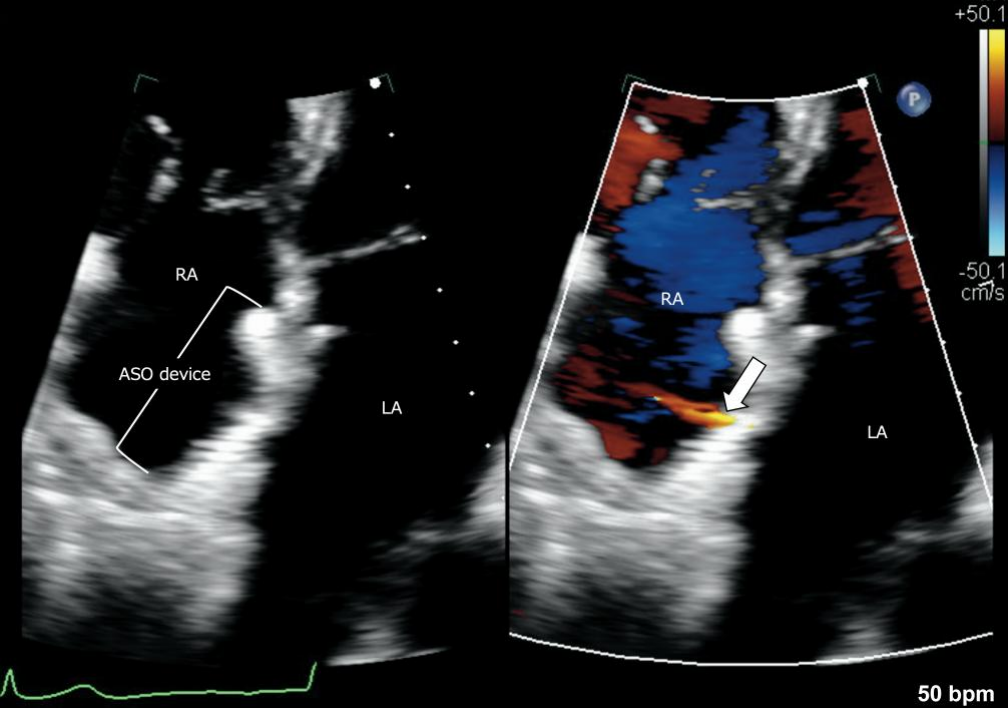
Residual shunt on transthoracic echocardiography. A transthoracic echocardiographic image delineating a residual interatrial shunt within 2 months after ASO device puncture. The residual shunt flow (arrow) was minimal in size and haemodynamically insignificant. ASO, atrial septal occluder; LA, left atrium; RA, right atrium.

Follow-up data are summarized in *Table 5*. During median follow-up of 22.0 months (IQR 7.0–69.5), AF/AT recurrence occurred in 9 patients (33.3%). Immediately after the procedure, all patients demonstrated a small residual interatrial shunt, all of which were tiny and haemodynamically insignificant (*Figure 7*, Supplementary material online, *Video S8*). Regardless of the puncture site, the detection rate progressively decreased over time, and among patients with available follow-up at 2 years, no residual shunt was observed. At each time point, there was no significant difference in the prevalence of residual shunt according to the puncture site. No major cardiovascular events, including tamponade, device dislodgement, embolization, heart failure hospitalization, or cardiovascular death, occurred.

**Table 5:** Follow-up data

Variables	Overall	Native septum-puncture group	ASO device–puncture group	*P* value
	*n* = 27	*n* = 17	*n* = 10	
Follow-up period after ablation, months	22.0 (7.0–69.5)	23.0 (7.0–71.0)	16.0 (6.5–69.0)	0.796
AF/AT recurrence, *n*	9 (33.3)	6 (35.3)	3 (30.0)	0.802
Residual interatrial shunt, *n*				
Immediate	27/27 (100)	17/17 (100)	10/10 (100)	1.000
2 months	10/23 (43.5)	5/13 (38.5)	5/10 (50.0)	0.685
6 months	5/25 (25.0)	3/12 (25.0)	2/8 (25.0)	1.000
1 year	2/18 (11.1)	2/11 (18.2)	0/7 (0)	0.497
2 years	0/17 (0)	0/11 (0)	0/6 (0)	1.000
Major cardiovascular events	0 (0)	0 (0)	0 (0)	1.000

Abbreviations are as in *Tables 1* and *2*.

*P* values for AF/AT recurrence are based on the log-rank test.

Major cardiovascular events included cardiac tamponade, device dislodgement, embolization, hospitalization for heart failure, and cardiac death.

## Discussion

This study demonstrated that TSP and catheter ablation can be safely and feasibly performed in patients with ASO devices. Additionally, we described the procedure for device puncture point by point in detail. Successful LA access was achieved in 27 of 28 sessions (96.4%), and all completed without major complications.

The decision to perform TSP through the native septum or the ASO device represents a key procedural consideration. Consistent with previous reports,^9^ we observed a tendency for device puncture to be required in cases with larger ASO device size. Although the small sample size precluded statistical significance, all PFO cases in our study were successfully accessed through the native septum. This finding may be attributed to the smaller device sizes typically used for PFO closure. In addition to device size, anatomical features such as defect location appeared to play an important role. Notably, patients with posterior or IVC rim deficiency required device puncture in all cases, irrespective of disc diameter. Taken together, these findings suggest that the feasibility of native septum puncture is determined not only by device dimensions but also by detailed septal anatomy and patient body habitus.

When device puncture was required, puncture area selection was the key determinant of procedural success. Targeting the waist region, where nitinol mesh is sparse and the number of layers minimized, combined with balloon-assisted sheath advancement (the ‘deflate-and-dive technique’), provided a high success rate while maintaining safety. The use of a mechanical needle was advantageous for guidewire passage and subsequent balloon dilation. One unsuccessful case involved a small wire-mesh-type occluder, likely due to denser mesh structure at the intended puncture site.

Although there is no established consensus regarding the optimal timing of TSP after ASO device implantation, it is generally advisable to perform the procedure only after the occluder device has become well endothelialized and firmly anchored to the septum, minimizing the risks of thromboembolism or device dislodgement. In our experience, a waiting period of at least 6 months—preferably 1 year—is recommended before attempting TSP. Because the endothelialization process may vary among patients, particular caution should be exercised in individuals receiving corticosteroid therapy or in elderly patients. In addition, anatomical factors such as the original defect morphology and rim deficiency should be carefully reviewed before the procedure. Patients with IVC rim deficiency or multiple rim defects may have a higher risk of device instability, and multidisciplinary discussion between electrophysiologists and cardiologists experienced in ASO device implantation is essential to ensure procedural safety.

Although TSP through an ASO device presents technical challenges—particularly in the absence of a native septal window—our results show these can be overcome with careful preprocedural planning and multimodality imaging guidance using TEE, CCT, and ICE. In the ASO device-puncture group, LA access time was prolonged, fluoroscopy time was longer, and total procedure time also tended to be longer. These differences may partly be attributed to the additional steps involved in obtaining LA access through the device.

No major procedural complications occurred, and residual shunts were low and transient, resolving spontaneously in all cases. These findings indicate that careful device puncture does not cause clinically significant long-term shunting. Variations in shunt detection rates among studies may reflect differences in echocardiographic resolution or follow-up protocols.

Long-term arrhythmia recurrence was similar between groups, suggesting that device puncture does not compromise ablation efficacy or safety. As the number of patients with transcatheter ASD closure grows, the need for AF ablation in this population will increase, underscoring the clinical relevance of these findings.

This study provides practical insights into puncture route selection, device traversal techniques, and procedural outcomes in patients with ASO devices. Despite its single-centre retrospective design, our findings support the feasibility and safety of AF ablation after ASO implantation and may aid electrophysiologists in procedural planning.

### Procedural tips for transseptal puncture through ASO devices

- **Imaging:** Use CCT to assess the availability of a puncturable native septum; if the septum is entirely covered by the device or native access is unsuccessful, proceed with direct device puncture.
- **Target:** Select the ASO waist (off-centre from the hub) as the optimal puncture site to minimize the number of mesh layers traversed.
- **Tool:** Utilize a mechanical needle to leverage its distal tip lumen, which facilitates the subsequent delivery of 0.014-inch-compatible balloons for ASO device dilation.
- **Manoeuver:** Apply the ‘deflate-and-dive’ technique, advancing the outer sheath in precise synchrony with balloon deflation to overcome the rapid elastic recoil of the nitinol mesh.

### Limitations and future directions

This single-centre retrospective study included a small sample size (28 sessions), limiting generalizability and power for rare complications. The study population consisted of referred patients deemed suitable for TSP, introducing selection and referral bias. Operator experience may also have influenced outcomes.

Patient heterogeneity existed in underlying pathology (ASD vs. PFO), AF type, prior ablation history, and device characteristics, which may have affected results. Procedural details such as sheath type, balloon size, staged dilation, and use of steerable sheaths were not fully standardized, and a learning-curve effect cannot be excluded. Arrhythmia recurrence was based on clinically documented episodes, and varying monitoring intensity may have led to underdetection of asymptomatic recurrences. Residual shunts were primarily assessed by TTE, potentially underestimating very small defects. Long-term device integrity, including mesh fatigue or deformation, was not systematically evaluated.

Future multicentre prospective registries are warranted to confirm these findings and to standardize imaging and procedural protocols. Such efforts will enable development of predictive models for feasible puncture sites based on detailed anatomical features such as disc diameter, defect location, and rim morphology. Larger studies are needed to evaluate rare complications, learning curves, radiation exposure, and long-term device durability. Integration of patient-reported outcomes and cost-effectiveness analyses will further clarify the comprehensive value of AF ablation in patients with ASO devices.

## Supplementary Material

euag071_Supplementary_Data

## Data Availability

The data underlying this article cannot be shared publicly due to ethical and privacy considerations. The data will be shared on reasonable request to the corresponding author.
